# A scoping review of statistical methods for the analysis of method comparison studies with repeated measurements of clinical data

**DOI:** 10.1186/s12874-026-02824-1

**Published:** 2026-03-11

**Authors:** Karine Brousseau, Victoria Ivankovic, Tori Lenet, Daniel I. McIsaac, Tim Ramsay, Dean A. Fergusson, Guillaume Martel

**Affiliations:** 1 School of Epidemiology and Public Health, Faculty of Medicine, University of Ottawa, Ottawa, ON Canada; 2 Ottawa Hospital Research Institute, Ottawa, ON Canada; 3 Liver and Pancreas Unit, Department of Surgery, The Ottawa Hospital, Ottawa, ON Canada; 4 Department of Anesthesiology and Pain Medicine, The Ottawa Hospital, Ottawa, ON Canada

**Keywords:** Bland-Altman, method comparison, repeated measurements, scoping review, statistical methods, continuous data

## Abstract

**Background:**

Method comparison studies are conducted to examine the level of agreement between two instruments measuring physiological continuous parameters. The inclusion of repeated measurements in such studies poses additional challenges. The Bland & Altman limits of agreement (LOA) approach has been adapted to account for the correlation between repeated measurements and is widely used in method comparison studies. Alternate statistical methods are not always appropriate for the analysis of such data, and there is a paucity of evidence and guidelines pertaining to statistical methods that inform the analysis of method comparison studies that include repeated measurements. This scoping review aimed to identify methodological publications that propose statistical methods to inform the analysis of method comparison studies that include repeated measurements of continuous clinical data and that may be compared with the LOA method.

**Methods:**

Six online databases were searched from inception to November 2022 using a peer-reviewed search strategy. Searching of grey literature and books, as well as backward citation searching were performed to identify additional sources of evidence. Screening and data abstraction were done by two independent reviewers. Results were synthesized narratively.

**Results:**

Twenty-nine publications were included in this review. Thirty-two independent statistical methods were identified from the included publications, including variants of the LOA method. Four included publications compared findings from different versions of the LOA method. Four different approaches to handling repeated measurements in the context of method comparison studies were identified and were used to group findings from the included publications. Reported strengths and limitations of the LOA method were summarized.

**Conclusion:**

This scoping review provides a synthesis of existing statistical approaches to inform the analysis of method comparison studies with repeated measurements of clinical data, as well as how the various statistical methods perform when compared with various version of the LOA method. Based on the findings, it is generally advisable to consider using adjusted LOAs or modified mixed-effect LOAs in analyzing method comparison studies with repeated measurements.

**Trial registration:**

The protocol was registered on Open Science Framework (https://osf.io/4p8ut).

**Supplementary Information:**

The online version contains supplementary material available at 10.1186/s12874-026-02824-1.

## Introduction

Method comparison studies are needed to examine agreement between new diagnostic methods proposed to measure continuous clinical data and a gold standard method [1]. For the analysis of method comparison studies, Bland & Altman (B&A) originally proposed the limits of agreement (LOA) method [1–4], providing an estimate of the range within which the difference in measurements from two different methods is expected to be, thus quantifying the extent of disagreement between methods [1]. The LOA method is widely accepted and utilized for the analysis of method comparison studies [5].

B&A have emphasized the importance of capturing repeated measurements within individual participants (i.e., multiple measurements of the same physiological parameter) in method comparison studies [1–3], whereby an interaction between subjects and methods needs to be accounted for [1, 2]. Importantly, repeated measurements allow for the assessment of within-subject variability (repeatability) for each method, which fundamentally constrains the degree of agreement that can be achieved between methods. Without repeated measurements, it is not possible to distinguish disagreement between methods from poor repeatability of one or both methods. While the LOA method has been well adapted for repeated measurements, other proposed statistical methods may or may not be appropriate for the analysis of method comparison studies with repeated measurements, making the identification and understanding of alternate statistical methods that are appropriate for this study design challenging. To our knowledge, there are no guidelines to inform this type of analysis.

This review sought to identify methodological papers discussing findings from various statistical methods to inform the analysis of method comparison studies with repeated measurements of continuous clinical data that may be compared with the LOA method. A scoping review framework was chosen to map and identify available evidence from varied sources [6–8].

## Methods

This scoping review is reported following the Preferred Reporting Items for Systematic Review and Meta-Analysis (PRISMA) extension for scoping reviews (PRISMA-ScR) [9]. A checklist can be found in Supplemental files. This review was conducted as a scoping review because its primary objective was to map and characterize the range of statistical methods used in method comparison studies with repeated measurements, rather than to quantitatively synthesize evidence or evaluate the performance of specific methods. Although a comprehensive search strategy was employed, consistent with best practice for scoping reviews, the intent was descriptive and conceptual rather than evaluative.

### Protocol and registration

Prior to initiating the review process, a protocol was created following the PRISMA extension for protocols (PRISMA-P) [10] and best methodological practices for scoping reviews [6, 8, 9, 11, 12], accessible via Open Science Framework (https://osf.io/4p8ut).

### Eligibility criteria

Eligibility criteria were determined following the Population, Concept, Context framework [11]. This review aimed to identify methodological reports reporting results from statistical methods proposed to analyse method comparison studies with continuous and repeated clinical data. Results from an LOA analysis [1–3] must have been available for comparison with the results from alternate statistical methods reported.

The dataset used to demonstrate the statistical methods must have been obtained or generated in a human population, in the context of a method comparison study with continuous and repeated measurements. The dataset may have been obtained from a past clinical study (secondary use), obtained for the purpose of demonstrating the statistical methods (primary use), or from simulated human clinical data. Methods being examined may have been any type of diagnostic tool, device, rater, instrument, or observer being compared for validation of any given clinical parameter measured in a continuous fashion. The term “method” will be used exclusively in this paper to encompass all those concepts.

Exclusion criteria consisted of using a dataset of non-clinical data or that lacked repeated measurements, and articles that did not report the quantitative results from statistical analyses or from an LOA analysis. There were no restrictions on language and date. Screened full-text citations in languages other than English or French were translated using Google Translate.

### Search strategy

A comprehensive search strategy was developed with a research librarian and peer reviewed following PRESS guidelines [13] and can be accessed in Supplemental files.

### Information sources

The final search strategy was conducted in Ovid Medline, Ovid EMBASE, CINAHL (EBSCO), Web of Science, and ProQuest Databases from inception to November 7, 2022, consistent with the study protocol and to allow sufficient time for screening, synthesis, and manuscript development. Forward citation searching was performed using Scopus to identify potentially eligible articles that have cited the B&A 2007 article [2], focusing on the LOA method adapted for repeated measures, from the date of publication to November 25, 2022.

Grey literature and books were systematically searched using Google and Google Books, respectively, with multiple small strings of key words adapted from our main search strategy. For each string, the first five search pages were screened. If at least one of the articles was identified as eligible, the sixth page was screened and so forth, until no potentially eligible articles were found within a search page. The grey literature was further examined by searching and posting on Stack Exchange - Cross Validated (https://stats.stackexchange.com, published November 29, 2022).

Backward citation searching was conducted to identify other potentially eligible reports within the references of included sources of evidence.

### Selection of sources of evidence

**Fig. 1:**
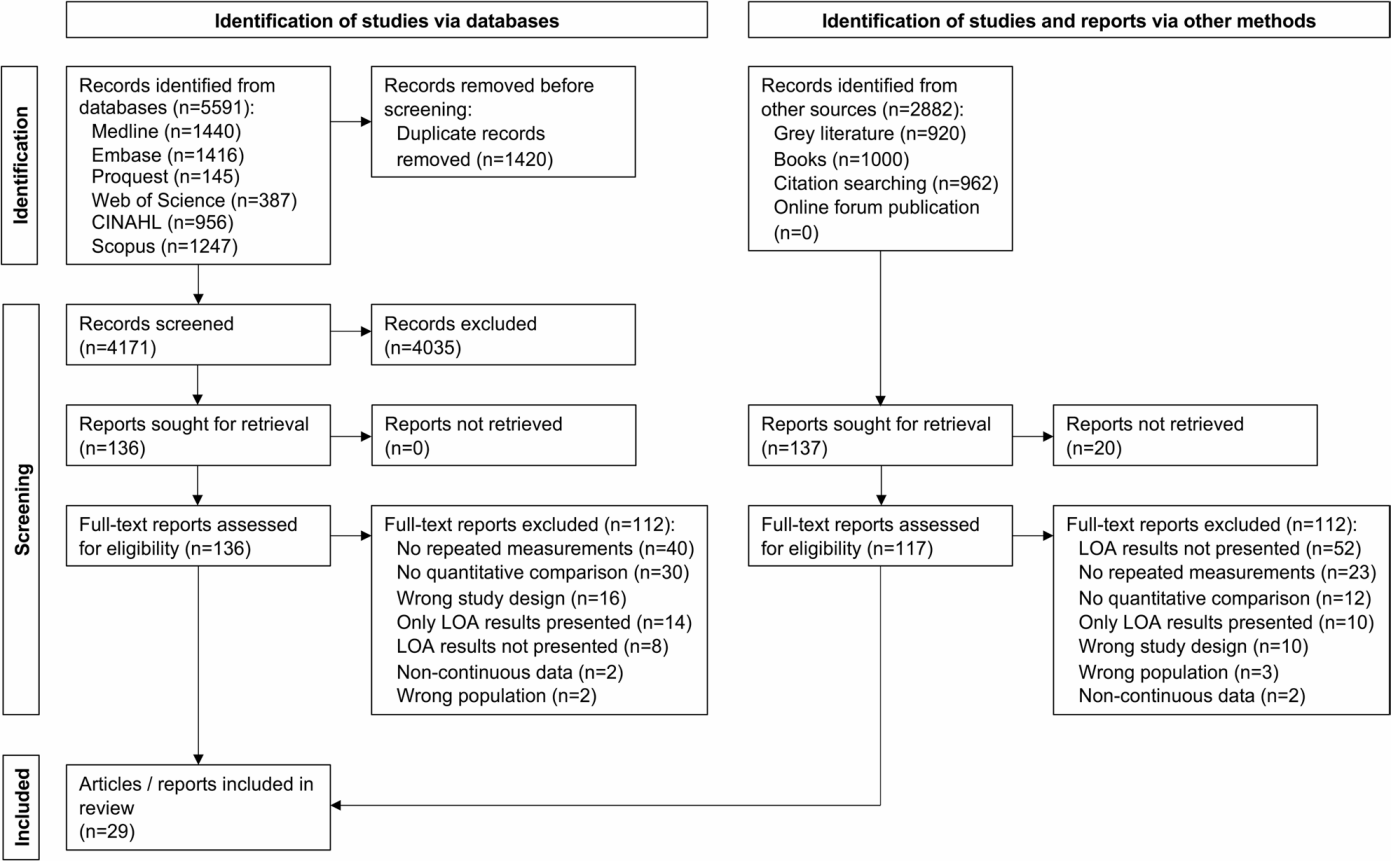
– PRISMA flow diagram

Identified reports were imported in Covidence (Covidence, Melbourne, Australia), duplicate reports were removed. Titles and abstracts screening and full-text review identified in database searches (Fig. 1) were screened in duplicate by two independent reviewers (KB, TL). Citations and reports identified from other methods were screened at each step by one reviewer (KB or TL). Conflicts were resolved by consensus, overseen by the senior authors (GM, DF).

### Data collection process

A data collection form was created for this review. A *post-hoc* decision was made to conduct data collection collaboratively between two reviewers (KB, TL). It was reasoned that this modification would increase efficiency and accuracy by discussing the various items and any arising conflicts while data charting.

### Data items

The following characteristics were collected from included publications: author names, country of the corresponding author, journal, funding, and type of publication. Information about the dataset used to conduct analyses and generate quantitative findings was also recorded. This included information on the context in which the dataset was created (primary use, secondary use, or simulation) and the population under study (eligibility criteria, mean age, sex distribution). Information concerning the sample size, the range of repeated measurements per participant, and the total sample of pairwise comparisons (e.g., 20 participants with 2 measurements each for all methods being compared, yielding 40 pairwise method comparisons) were charted. Further dataset items were the diagnostic methods being compared, identifying the index tests being examined for accuracy, the primary outcome measured, and missing data. No assumptions were made to complete the above data items.

Finally, data concerning the findings of each statistical method being compared were charted. This included the quantitative findings from each statistical analysis, the conclusions regarding the agreement between methods from each statistical analysis, and the criteria used to determine agreement. When either the conclusion about agreement between methods or the agreement criteria used were not clearly reported in the publication, they were extrapolated from the available data given that this was an important outcome.

Finally, information concerning the adjustment for correlation between repeated measurements were extracted. When these data items were not reported, they were extrapolated using the available information. Reported strengths and limitations associated with each statistical method were charted.

### Synthesis of results

The characteristics of included articles and their underlying datasets and comparisons between the quantitative findings of various statistical methods were summarized. A list of all reported statistical methods was generated, with their associated incidence. Furthermore, the frequency with which the conclusions about agreement from each statistical analysis matched with those from the LOA analysis was computed. Quantitative meta-analysis was not possible, owing to the variety of outcomes measured and of statistical methods being presented. Included publications were grouped based on the adjustments done for repeated measurements. Finally, an overview of the strengths and limitations of the LOA method was produced.

### Statistical methods presented as alternatives to the LOA method

Three versions of the LOA method were identified and described in the included sources of evidence. (1) The standard LOA method proposed by B&A, which can be used in the absence of repeated measurements, when only one measurement pair is used [3], or in cases when the mean of the repeated measurements is used for the LOA analysis, thus yielding only one pairwise comparison [1, 2]. (2) The adjusted LOA method, adjusting for the correlated nature of the repeated measurements, as proposed by B&A^1,2^. Authors have also referred to this approach as using fixed effect principles [14–16]. (3) Modified LOA methods, being alternate versions of the LOA method, using random effects models to account for repeated measurements [14–16].

### Critical appraisal of individual sources of evidence

As planned, risk of bias assessment and critical appraisal of individual sources of evidence was not done [6, 7].

## Results

### Selection of sources of evidence

The search strategy identified 5591 citations from major databases, in addition to 2882 from grey literature. After de-duplication and screening, the final review included 29 full-text studies (Fig. 1).

### Characteristics of sources of evidence

Publication characteristics and the specifications of the underlying datasets used are presented in Table 1. The number of participants in the included studies ranged from 6 to 1035. The total number of pairwise comparisons per participant ranged from 1 to 60,000. The type of methods that were compared in the context of the underlying method comparison studies were devices (*n* = 20) [1, 3, 14–32], questionnaires (*n* = 4) [33–36], and observers (*n* = 4) [37–40].

**Table 1:** Study and dataset characteristics

Study author(s), year, Country, publication type	Type of dataset used*	Sample size (participants/ comparisons)***	Measurements per rater, per participant / total number of comparisons	Outcome(s) measured (units)	Number and type of raters****	Reference standard (RS)	Index test(s) (IT)
Alanen [17], 2010, Finland, Scientific article	Secondary use of a generated dataset [3]	17 / 34	2 / 34	Peak expiratory flow rate (PEFR) (L/min)	2 devices	Wright peak flow meter	Mini Wright peak flow meter
Ambrosini et al. [33], 2001, Australia, Scientific article	Original and generated dataset	76 / 76	2 IT, 4 RS / 152	Dietary beta-carotene (mg) / Dietary retinol (mcg)	2 questionnaires	Mean of four 1-week diet records	Two Food Frequency Questionnaires (FFQ)
Anvari et al. [37], 2017, USA, Scientific article	Secondary use of a clinical dataset [43]	35 / 35	2 / 70	Thyroid nodule tissue stiffness (kPa)	2 observers	Not applicable (NA)	NA
Armstrong et al. [38], 2006, USA, Scientific article	Secondary use of a clinical dataset**	6 / 24	4 / 24	Urethrovesical junction (UVJ) position and mobility, using a rectangular coordinate method (mm)	2 observers (R1, R2)	NA	NA
		24 / 24	4 / 96		2 observers (R1, R3)	NA	NA
Batterham et al. [34], 2016, Australia, Scientific article	Original and simulated dataset	80 / 80	1 IT, 3 RS / 240	Mean Iodine intake (mcg)	2 questionnaires	Mean of three 24-hour dietary recall	FFQ
Bland & Altman, 1999 [1], UK, Scientific article	Secondary use of a clinical dataset [44]	85 / 255	3 / 255	Systolic blood pressure (SBP) (mmHg)	2 devices	Sphygmomanometer (by observer “J”)	Machine “S”
Bland & Altman, 1986 [3], UK, Scientific article	Original and generated dataset	17 / 17	2 / 34	PEFR (L/min)	2 devices	Wright peak flow meter	Mini Wright peak flow meter
Cecconi et al. [18], 2009, Italy, Scientific article	Not reported (NR)	20 / 20	1 RS, 4 IT / 80	Cardiac output (L/min)	2 devices	Thermodilution	Intermittent thermodilution
Choudhary [19], 2008, USA, Scientific article	Secondary use of a clinical dataset [1, 45]	12 / 60	3–6 / 60	Left ventricular ejection fraction (LVEF) (%)	2 devices	Radionuclide ventriculography	Impedance cardiography
Choudhary and Nagaraja [46], 2017, USA, Book chapter	Secondary use of a clinical dataset**	61 / 177	1–3 / 177	Blood oxygen saturation (%)	2 devices	Co-oxymetry	Pulse oxymetry
Euser et al. [39], 2008, Netherlands, Scientific article	Secondary use of a clinical dataset [47]	4 / 4	2 / 8	Biceps skinfold thickness (mm)	13 observers	NA	NA
Francq and Govaerts [21], 2015, Belgium, Scientific article	Secondary use of a clinical dataset [1, 44]	85 / 255	3 / 255	SBP (mmHg)	2 devices	Sphygmomanometer (by observer “J”)	Machine “S”
Haghayegh et al.[35], 2020, USA, Scientific article	Original and simulated dataset	80 / 80	3 RS, 1 IT / 240	Mean intake of Iodine (mcg)	2 questionnaires	Mean of three 24-hour dietary recall	FFQ
Kim and Lee [22], 2022, USA, Scientific article	Secondary use of a generated dataset [3]	17 / 17	2 / 34	PEFR (L/min)	2 devices	Wright peak flow meter	Mini Wright peak flow meter
Lai and Shiao [23], 2005, USA, Scientific article	Secondary use of a clinical dataset [48]	2 / 625	NR / 625	Foetal haemoglobin percentage levels (%)	2 devices	Percent fractional oxyhemoglobin (HbO_2_)	Percent functional oxyhemoglobin (SO_2_)
Lombard et al. [36], 2015, South Africa, Scientific article	Original and generated dataset	47 / 47	4 RS, 1 IT / 188	Energy intake (kJ)	2 questionnaires	Mean of four 24-hour dietary recall	FFQ
Lorne et al.[24], 2018, France, Scientific article	Secondary use of a clinical dataset [49]	24 /199	NR / 199	Cardiac output (L/min)	6 devices	Pulmonary artery thermodilution	5 arterial pulse contour techniques (Wesseling method, LiDCO, PiCCO, Hemac method, Modelflow)
Myles and Cui [15], 2007, Australia, Editorial	Secondary use of a clinical dataset [50]	20 / 144	7–8 / 144	Oxygen consumption (mL/min)	2 devices	Inspired gas analysis (GVO_2_)	Reverse Fick method (FVO_2_)
Parker et al. [16], 2020, Canada, Scientific article	Secondary use of a clinical dataset [14, 51]	21 / 385	15–19 / 385	Respiratory rate (breaths/ minute)	2 devices	Standard respiratory rate monitor (Oxycon mobile)	Chest-band
Parker et al. [14], 2016, Scotland, Scientific article	Secondary use of a clinical dataset [51]	21 / 385	9–19 / 385	Respiratory rate (breaths/ minute)	6 devices	Standard respiratory rate monitor (Oxycon mobile)	Five commercially available respiratory rate monitors: Camera, photoplethysmography (PPG), Impedance, Accelerometer, Chest-band
Quiroz and Burdick [25], 2009, USA, Scientific article	Secondary use of a generated dataset [3]	17 / 34	2 / 34	PEFR (L/min)	2 devices	Wright peak flow meter	Mini Wright peak flow meter
Rankin and Stokes [40], 1998, UK, Scientific article	Original and generated dataset	10 / 10	4 GR, 1 MS / 40	Cross-section area of anterior tibial muscle group (cm^2^)	2 observers (GR, MS)	NA	NA
Roy [26], 2009, USA, Scientific article	Secondary use of a clinical dataset [1, 44]	85 / 255	3 / 255	SBP (mmHg)	2 devices	sphygmomanometer (by observer “J”)	Machine “S”
	Secondary use of a generated dataset [3]	17 / 34	2 / 34	PEFR (L/min)	2 devices	Wright peak flow meter	Mini Wright peak flow meter
	Secondary use of a clinical dataset [1, 45]	12 / 60	3–6 / 60	LVEF (%)	2 devices	Radionuclide ventriculography	Impedance cardiography
Saugel et al. [27], 2018, Germany, Scientific article	Secondary use of a clinical dataset [52]	1035 / 1035	1–60,000 / NR	Arterial systolic blood pressure	2 devices	arterial catheter	oscillometry
				Mean arterial blood pressure	2 devices	arterial catheter	oscillometry
Schluter [28], 2009, Australia, Scientific article	Secondary use of a clinical dataset [1, 44]	85 / 255	3 / 255	SBP (mmHg)	2 devices	sphygmomanometer (by observer “J”)	Machine “S”
Stevens et al. [29], 2018, USA, Scientific article	Secondary use of a clinical dataset [1, 44]	85 / 255	3 / 255	SBP (mmHg)	2 devices	sphygmomanometer (by observer “J”)	Machine “S”
Stevens et al. [30], 2017, Canada, Scientific article	Secondary use of a clinical dataset [1, 44]	85 / 255	3 / 255	SBP (mmHg)	2 devices	sphygmomanometer (by observer “J”)	Machine “S”
Taffé [32], 2021, Switzerland, Scientific article	Original and simulated dataset	100 / NR	10–20 / NR	“Simulating data from a uniform distribution having values between 20 and 40.”	2 simulated devices	Simulated device “Y1”	Simulated device “Y2”
Taffé [31], 2020, Switzerland, Scientific article	Secondary use of a clinical dataset [1, 44]	85 / 255	3 / 255	SBP (mmHg)	2 devices	sphygmomanometer (by observer “J”)	Machine “S”

*Abbreviations*: *FFQ* Food Frequency Questionnaire, *GR* Gold-standard rater, *IT* Index test, *kPa* kilopascals, *L/min* Iitres per minute, *LVEF* Left ventricular ejection fraction, *mcg* micrograms, *mg* Milligrams, *mm* Millimetres, *mmHg* Millimetres of mercury, *NA* Not applicable, *NR* Not reported, *PEFR* Peak expiratory flow rate, *PPG* Photoplethysmography, *RS* Reference standard, *SBP* Systolic blood pressure, *UVJ* Urethrovesical junction

*Datasets may be *original*, meaning that it was created for the purpose of the presented paper, or the authors could have used an existing dataset for *secondary use*. The dataset may be *clinical*, *generated*, or *simulated*. A clinical dataset was created for clinical research (i.e., method comparison study in a given population) and is now used in the context of the presented article. A generated dataset was collected within a random set of individuals for the purpose of demonstrating statistical methods as opposed to aiming to examine rater accuracy. A simulated dataset was not captured in humans, but generated via simulation as though they were clinical measurements done on humans

**Reference to original dataset not reported

***Sample size represents the total number of participants and observations used for analysis, which may not be equal to the total number of comparisons, depending on how they handle the repeated measurements

****This includes the raters that were used for analysis. More raters may have been measured in the dataset, but not analyzed within the presented study

### Results of individual sources of evidence

Quantitative findings from statistical methods presented in included publications are summarized in Table 2. Among all included studies, 10 produced divergent conclusions about agreement based on the statistical methods that were used. In the presence of divergent conclusions between LOA analysis and alternate statistical methods, the LOA analysis concluded that the underlying methods disagreed in all but one study^18^, whereas alternate statistical methods yielded agreement [3, 16, 17, 22, 24, 26, 33,36, 38].

**Table 2:** Quantitative results

Study author(s)	Statistical methods used	Quantitative results**	Conclusion about agreement	Agreement criteria used
Alanen	Standard Limits of agreement (LOA)^3^	LOA_L_: -79.7 (95%CI -114.3, 45.1) LOA_U_: 75.5 (95%CI 40.9, 110.1)^3^	No agreement	Limits within ±10 L/min^3^
	Structural equations (SEM) approach	Fit of the final model: χ^2^ = 7.50 Degrees of freedom (df) = 11	Agreement	Acceptance of the null hypothesis
Ambrosini et al.	Standard LOA	β-carotene: LOA_L_: 50%, LOA_U_: 447%	No agreement	100% represents ideal agreement
		Retinol: LOA_L_: 11%, LOA_U_: 349%	No agreement	
	Pearson correlation coefficient (PCC)	β-carotene: 0.36 (95%CI: 0.14–0.54)	Agreement	95% CI not including the value “0” is statistically significant
		Retinol: 0.51 (95%CI: 0.32–0.66)	Agreement	
	Mean agreement	β-carotene: 149% (95%CI: 132–170)	No agreement	95% CI not including the value “100%” is statistically significant, 100% represents ideal agreement
		Retinol: 63% (95%CI: 52–77)	No agreement	
Anvari et al.	Standard LOA	LOA_L_: -9.9, LOA_U_: 11	Agreement	NR
	Intraclass correlation coefficient (ICC)	Single measurement: 0.872 (95% CI: 0.763–0.933),	Agreement	ICC > 0.70
		Average measurements: 0.932 (95% CI: 0.865–0.965)	Agreement	
Armstrong et al.*	Standard LOA	R1/R2: Kegel D_x_: -9, 8, Kegel D_y_: -6, 14	No agreement on both axes	Limits within ±2 mm
		R1/R3: Kegel D_x_: -5, 5, Kegel D_y_: -4, 5	No agreement on both axes	
	PCC	R1/R2: Kegel D_x_: 0.87, Kegel D_y_: 0.70	Agreement for D_x_, no agreement for D_y_	NR (Assumed PCC > 0.7)
		R1/R3: Kegel D_x_: 0.96, Kegel D_y_: 0.93	Agreement on both axes	
	T-test	R1/R2: Kegel D_x_: *P* value not significant (NS) Kegel D_y_: *P* < 0.001	Agreement for D_x_, no agreement for D_y_	*P* value < 0.05
		R1/R3: Kegel D_x_: *P* value NS Kegel D_y_: *P* value NS	Agreement on both axes	
Batterham et al.	Standard LOA	LOA_L_: -88.38 (95%CI -122.24, -54.52) LOA_U_: 83.82 (95%CI 49.96, 117.96)	Agreement based on point estimates only. No agreement based on 95%CI	NR (assumed limits within 90 to 100 mcg)
	Two one-sided t-test (TOST)	Equivalence margin (SEM) of 5 mcg: *t* upper: 0.55 (*p* = 0.291) *t* lower: -1.48 (*p* = 0.071)	No agreement	Both TOST *P*-values < 0.05 with 79 degrees of freedom, with a 90% CI of the mean difference within − 10.49 to 5.89 used for equivalence testing
		SEM of 10 mcg: *t* upper: 1.57 (*p* = 0.060) *t* lower: -2.50 (*p* = 0.007)	No agreement	
		SEM of 15 mcg: *t* upper: 2.69 (*p* = 0.006) *t* lower: -3.52 (*p* = 0.000)	Agreement	
		SEM of 10%: *t* upper: 2.06 (*p* = 0.021) *t* lower: -2.99 (*p* = 0.002)	Agreement	
	Paired t-test	*t* -0.465 (*p* = 0.643)	NR (assumed no agreement)	Assumed based on *P*-value
Bland & Altman, 1999 [1]	Standard LOA	LOA_L_: -54.7 (95%CI -61.9, -47.5) LOA_U_: 22.1 (95%CI 14.9, 29.3)	No agreement	Limits within ±10 mmHg
	Adjusted LOA	LOA_L_: -56.68 (95%CI -63.5, -49.9) LOA_U_: 25.44 (95%CI 18.7, 32.2)	No agreement	
	Non-parametric approach	Grade D (16% within 5 mmHg, 35% within 10 mmHg, 49% within 15 mmHg - fails to achieve Grade C)	No agreement	To achieve grade C: at least 40% measurements ≤5 mmHg, 65% ≤10 mmHg, 85% ≤15 mmHg
Bland & Altman, 1986 [3]	Standard LOA	LOA_L_: 79.7 (95%CI -114.3, -45.1) LOA_U_: 75.5 (95%CI 40.9, 110.1)	No agreement	Limits within ±10 L/min
	Correlation coefficient	*r* = 0.94 (*p* < 0.001)	“Measurements are linearly related”	Test of significance (*P* < 0.05)
Cecconi et al.	Standard LOA	LOA_L_: -2.7, LOA_U_: 2.3	Agreement	NR
	Coefficient of error (CE)	CE = 4% CV = 15% PE = 30%	No agreement, based on CE and CV alone, agreement based on PE	PE within ±30%
Choudhary, 2008 [19]	Adjusted LOA^1^	LOA_L_: -1.3521, LOA_U_: 2.7705^1^	NR^1^	NR^1^
	Tolerance interval	*U*_*x*_=2.33	*Poor agreement*	NR
Choudhary and Nagaraja, 2017 [20]	LOA (version not reported)	LOA_L_: -9.6, LOA_U_: 14.6	NR	NR
	Concordance correlation coefficient (CCC)	0.85 (lower one-sided 95% confidence bound: 0.80)	Weak agreement	NR
	Total deviation index (TDI) (0.90)	10.91 (upper one-sided 95% confidence bound: 12.14)	Weak agreement	NR
Euser et al.	Standard LOA	Log-transformed measurements: LOA_L_: -0.392, LOA_U_: 0.392 Ratio of 2 measurements: LOA_L_: 0.400, LOA_U_: 2.499 Difference between 2 measurements as a function of the mean X: LOA_L_: -0.85X, LOA_U_: 0.85X	NR	NR
	Coefficient of variation (CV)	Inter-observer CV = 12.5% Inter-observer CV of log-transformed data = 33.1%	No agreement, based on log-transformed data	NR
Francq and Govaerts	Adjusted LOA	Agreement interval (AI): LOA_L_: -25.440, LOA_U_: 56.679 XL-AI: LOA_L_: -29.871, LOA_U_: 61.110	No agreement	Limits within ±10 mmHg
	Correlated-errors-in-variables model (CEIV)	𝛽 tolerance interval: -6.030, 37.269 𝛽𝛾 tolerance interval: -6.757, 37.996	No agreement	
Haghayegh et al.	Adjusted LOA	Simulation 1: LOA_L_: -1127.14 (95%CI -1462.45, -791.84) LOA_U_: 55.36 (95%CI -279.95, 390.67) Simulation 2: LOA_L_: -2.869 (95%CI -5.652, -0.086) LOA_U_: 7.629 (4.846, 10.412)	NR	« when MDC (minimal detectable change) < MCIC (minimal clinically important change) and bias ≈ 0”
	ICC	Simulation 1: ICC_Absolute_: 0.855 (95%CI 0.60, 0.95) ICC_Consistency_: 0.99 (95%CI 0.997, 1.000) Simulation 2: ICC_absolute_: 0.977 ICC_consistency_: 0.990	NR	95%CI of ICC: < 0.5 = poor reliability 0.5–0.75 = moderate reliability 0.75–0.90 = good reliability > 90 = excellent reliability
Kim and Lee	Standard LOA	LOA_L_: -84.30, LOA_U_: 80.06	No agreement	NR
	CCC	0.943	Agreement	CCC > 0.75
	PCC	0.943	NR	NR
	Bias correction factor	0.999	NR	NR
Lai and Shiao	Standard LOA	LOA_L_: -2.48, LOA_U_: 4.54	No agreement	NR – visual inspection of the Bland & Altman plot
	Linear mixed model	Fixed effect parameter intercept: 2.5056 (*p* = 0.0054) Estimated parameter of the coefficient of fetal hemoglobin percent: -0.0263 (*p* < 0.0001) Random effect first-order autocorrelation parameter: *ρ* = 0.6978 (*p* < 0.0001)	No agreement	*P*-value < 0.05 associated with the estimated parameters
Lombard et al.	LOA (version not reported)	LOA_L_: -13,406, LOA_U_: 10,694 (*p* < 0.0001)	“Biased agreement”	*p* < 0.05 = no agreement
	Spearman Correlation	*r* = 0.26 (*p* < 0.0001)	Acceptable agreement based on *r* value, biased agreement based on *p* value	Good: ≥ 0.50; Acceptable: 0.20–0.49; Poor < 0.20
	Wilcoxon Signed Rank test	*P* > 0.05	Good agreement	Good: *p* > 0.05; Poor: ≤ 0.05
	Percentage difference (%)	-9.8%	Good agreement	Good: 0.0–10.9%; Acceptable: 11.0–20.0%; Poor: > 20.0%
	Cross classification (% in same tertile)	46.8%	Poor agreement	Good: ≥ 50%; Poor: < 50%
	Cross classification (% in opposite tertile)	19.2%	Poor agreement	Good: ≤ 10%, Poor: > 10%
	Weighted kappa statistic	0.2	Acceptable agreement	Good: ≥ 0.61; Acceptable: 0.20–0.59; Poor: < 0.20
Lorne et al.	Standard LOA [49]	Wesseling: LOA_L_: -0.80, LOA_U_: 1.26	No agreement	Limits within ±0.5
		LiDCO: LOA_L_: -1.55, LOA_U_: 1.20	No agreement	
		PiCCO: LOA_L_: -1.60, LOA_U_: 1.89	No agreement	
		Hemac: LOA_L_: -0.81, LOA_U_: 1.89	No agreement	
		Modelflow: LOA_L_: -0.74, LOA_U_: 0.74	No agreement	
	Interchangeability rate	Wesseling: Inclusion rate 76%	No agreement	≥ 95% = excellent ≥ 90% = good 75–90% = poor < 75% = not clinically relevant
		LiDCO: Inclusion rate 73%	No agreement	
		PiCCO: Inclusion rate 62%	No agreement	
		Hemac: Inclusion rate 86%	No agreement	
		Modelflow: Inclusion rate 93%, Interchangeability cut-off 4.80	Agreement	
Myles and Cui	Standard LOA	LOA_L_: -128, LOA_U_: 88	NR	NR
	Adjusted LOA	LOA_L_: -154, LOA_U_: 95	Unacceptable agreement	NR
	Modified (random effects) LOA	LOA_L_: -116, LOA_U_: 57	NR	NR
Parker et al., 2020 [16]	Standard LOA	-6.40 to 3.19	No agreement	Limits within ±5
	Adjusted LOA	-9.99 to 6.78	No agreement	
	Modified (mixed effect) LOA	-11.57 to 8.38	No agreement	
	Modified (mixed effect) LOA, with interactions	-11.86 to 9.30	No agreement	
	CCC	0.68 (95% CI 0.60 to 0.72)	“Slight” agreement	95%CI does not include the value of zero or negative values
	TDI	10.9 (95% CI 9.4 to 12.7)	No agreement	Clinically acceptable difference of ±5
	Coverage probability (CP)	0.63 (95% CI 0.56 to 0.70)	No agreement	≥ 95%
	Coefficient of individual agreement (CIA)	0.68 (95% CI 0.57 to 0.75)	No agreement	≥ 80%
	PCC	0.74 (95%CI 0.69, 0.78)	NR	NR
	Unadjusted CCC	0.72 (95%CI 0.67, 0.76)	NR	NR
Parker et al., 2016* [14]	Adjusted LOA	Camera (rate/ second): LOA_L_: -13.35, LOA_U_: 6.72	No agreement	Limits within ±10
		Accelerometer: LOA_L_: -8.74, LOA_U_: 4.38	Agreement	
	Modified (mixed effect) LOA	Camera (rate/ second): LOA_L_: -12.71 (95%CI -14.84, -11.42) LOA_U_: 6.30 (95%CI 5.00, 8.39)	No agreement	
		Accelerometer: LOA_L_: -8.63 (-9.45, -7.96) LOA_U_: 4.27 (95%CI 3.62, 5.21)	Agreement	
	Modified (mixed effect) LOA, after removing outliers	Camera (rate/ second): LOA_L_: -11.54 (95%CI -13.20, -10.40) LOA_U_: 5.24 (95%CI 4.10, 6.80)	No agreement	
		Accelerometer: LOA_L_: -7.91 (95%CI -8.75, -7.20) LOA_U_: 3.63 (95%CI 3.01, 4.64)	Agreement	
Quiroz and Burdick	Standard LOA [3]	LOA_L_: -79.7 (95%CI -114.3, -45.1) LOA_U_: 75.5 (95%CI 40.9, 110.1)	No agreement [3]	Limits within ±10 L/min^3^
	90% Generalized confidence intervals (GCI)	GCI_L_: -79.14, GCI_U_: 85.34	No agreement	At least 90% of the absolute difference is less than 10
Rankin and Stokes	Standard LOA	LOA_L_: -2.73, LOA_U_: 1.53	“Reasonable” Agreement	Limits within ±1.5
	ICC	0.92 (95% CI 0.72 to 0.98)	Agreement	NR
Roy	Adjusted LOA	SBP data^1^: LOA_L_: -56.68 (95%CI -63.5, -49.9) LOA_U_: 25.44 (95%CI 18.7, 32.2)	]No agreement []	Limits within ±10 mmHg^1^
		PEFR data^3^: LOA_L_: 79.7 (95%CI -114.3, -45.1) LOA_U_: 75.5 (95%CI 40.9, 110.1)	No agreement [3]	Limits within ±10 L/min^3^
		LVEF data^1^: LOA_L_: -1.3521, LOA_U_: 2.7705	NR^1^	NR^1^
	Linear mixed effect model (LME), based on Bonferroni adjusted p-values for variabilities	SBP data: Bias: *p* < 0.0001 Between-subject: *p* = 1.0 Within-subject: *p* < 0.0001	No agreement	p-values ≥ 0.05
		PEFR data: Bias: *p* = 1.0 Between-subject: *p* = 1.0 Within-subject: *p* = 0.8199	Agreement	
		LVEF data: Bias: *p* = 0.0612 Between-subject: *p* = 1.0 Within-subject: *p* = 1.0	Agreement	
Saugel et al.	Standard LOA	Systolic arterial pressure: LOA_L_: -43.9, LOA_U_: 56	No agreement	Bias ±5 mmHg and SD ±8 mmHg
		Mean arterial pressure: LOA_L_: -17.1, LOA_U_: 73.6	No agreement	
	Error grid analysis (EGA)	Systolic arterial pressure: risk levels A-E: 78%, 14%, 6%, 1%, and 1%	NR	NR
		Mean arterial pressure: risk levels A-E: 18%, 54%, 20%, 7%, and 8%	NR	NR
Schluter	Adjusted LOA [1]	LOA_L_: -56.68 (95%CI -63.5, -49.9) LOA_U_: 25.44 (95%CI 18.7, 32.2)	No agreement [1]	Limits within ±10 mmHg^1^
	Exchangeable multivariate hierarchical Bayesian model (HB1)	LOA_L_: -56.2, LOA_U_: 25.1	No agreement	NR
	Non-exchangeable multivariate hierarchical Bayesian model (HB2)	LOA_L_: -55.9, LOA_U_: 25.0	No agreement	NR
Stevens et al., 2018 [29]	Adjusted LOA [1]	LOA_L_: -56.68 (95%CI -63.5, -49.9) LOA_U_: 25.44 (95%CI 18.7, 32.2)	No agreement []	Limits within ±10 mmHg^1^
	Probability of agreement approach, using a clinically acceptable difference ±10 (heteroscedastic)	Probability of agreement never exceeds 0.3	No agreement	θ > 0.95
Stevens et al., 2017 [30]	Adjusted LOA	LOA_L_: -56.68 (95%CI -63.5, -49.9) LOA_U_: 25.44 (95%CI 18.7, 32.2) ^1^	No agreement	Clinically acceptable difference ±10 mmHg^1^
	Probability of agreement approach, using a clinically acceptable difference ±10 (homoscedastic)	Unconditional probability of agreement (θ) 0.799 (95%CI 0.61, 0.98)	No agreement	θ > 0.95
Taffé, 2021 [32]	LOA (version not reported)	LOA_L_: -19.6, LOA_U_: 15.3	NR	NR
	Bias, agreement, and precision plots	Assessment of agreement is done by visual inspection of a series of plots.	NR	Assessment of confidence bands in precision plot.
Taffé, 2020 [31]	Adjusted LOA [1]	LOA_L_: -56.68 (95%CI -63.5, -49.9) LOA_U_: 25.44 (95%CI 18.7, 32.2)	No agreement [1]	Limits within ±10 mmHg^1^
	Bias, agreement, and precision plots	Assessment of agreement is done by visual inspection of a series of plots.	No agreement	Visual inspection of the precision plot (confidence bands do not overlap)

*Abbreviations*: *AI* Agreement interval, *B&A* Bland and Altman, *CCC* Concordance correlation coefficient, *CE* Coefficient of error, *CEIV* Correlated-errors-in-variables model, *CI* Confidence interval, *CIA* Coefficient of individual agreement, *CP* Coverage probability, *CV* Coefficient of variation, *df* Degrees of freedom, *EGA* Error grid analysis, *GCI* Generalized confidence interval, *HB* Hierarchical Bayesian model, *ICC* Intraclass correlation coefficient, *IT* Index test, *LOA* Limits of agreement, *LOAL* Lower limit of agreement, *LOAU* Upper limit of agreement, *LME* Linear mixed-effects model, *MDC* Minimal detectable change, *MCIC* Minimal clinically important change, *NA* Not applicable, *NR* Not reported, *PCC* Pearson correlation coefficient, *PE* Percentage error, *RS* Reference standard, *SBP*, Systolic blood pressure, *SEM*, Standard error of measurement, *TDI* Total deviation index, *TOST* Two one-sided test, *UVJ* Urethrovesical junction

*Not all results from this paper are presented if many had similar conclusions. Results presented were chosen to show variety in agreement conclusions

**When a reference is provided with LOA results, LOA results were extracted from a different source

### Synthesis of results

Twenty-five publications included in this review have used only one version of the LOA methods, whereas four publications compared findings obtained from multiple versions of the LOA method. [1, 14–16]. Eighteen publications reported findings using the standard LOA method [1, 3, 15–18, 22–27, 33, 34, 37–40].

Eleven publications reported findings using the adjusted LOA method [1,14–16, 19, 21, 28–31, 35]. Three publications presented variations of the LOA method (modified LOA), using random effects models to adjust for the correlated nature of the repeated measurements [14–16]. Three publications did not report on the method used to generate LOA findings, nor could it be deduced from the full-text [20, 32, 36].

#### Adjustments for correlation with repeated measurements

Publications were separated into five different groups, based on the methods used to adjust for repeated measurements (Table 3). One publication did not report how the repeated measurements were used for any of the statistical analyses [36].

**Table 3:** Methods for handling repeated measurements

Study authors	Using one measurement only, excluding the repeated measures from the analyses	Considering all data as independent, ignoring correlated nature of the repeated measures	Using the means of the repeated measurements	Adjusting for correlation between repeated measurements	Not reported
1. Comparison between various versions of the LOA					
Bland & Altman, 1999* [1]	Standard LOA			Adjusted LOA	
Myles and Cui*	Standard LOA			Adjusted LOA, Modified LOA	
Parker et al., 2020* [16]	Standard LOA			Adjusted LOA, Modified LOA	
Parker et al., 2016 [14]				Adjusted LOA, Modified LOA	
2. Alternate statistical methods adjusted for repeated measurements versus adjusted LOA					
Choudhary, 2008** [19]				Adjusted LOA, tolerance interval	
Francq and Govaerts				Adjusted LOA, Correlated-errors-in-variables model (CEIV)	
Haghayegh et al.				Adjusted LOA, Intraclass correlation coefficient (ICC);	
Schluter**				Adjusted LOA, Exchangeable multivariate hierarchical Bayesian model (HB1); Non-exchangeable multivariate hierarchical Bayesian model (HB2)	
Stevens et al., 2018** [29]				Adjusted LOA, Probability of agreement approach, using a clinically acceptable difference ±10 (heteroscedastic)	
Stevens et al., 2017** [30]				Adjusted LOA Probability of agreement approach, using a clinically acceptable difference ±10 (homoscedastic)	
Taffé, 2020** [31]				Adjusted LOA, Bias, agreement, and precision plots	
3. Alternate statistical methods adjusted for repeated measurements versus standard LOA					
Alanen**	Standard LOA			Structural equations(SEM)	
Cecconi et al.			Standard LOA	coefficient of error (CE); coefficient of variation (CV); percentage error (PE)	
Choudhary and Nagaraja, 2017 [46]				Total deviation index (TDI); Concordance correlation coefficient (CCC)	LOA (version not reported)
Lai and Shiao		Standard LOA		Linear mixed model	
Quiroz and Burdick**	Standard LOA			90% Generalized confidence intervals (GCI)	
Roy**	Standard LOA			Linear mixed effect model (LME), based on Bonferroni adjusted p-values for variabilities	
Taffé, 2021 [32]				Bias, agreement, and precision plots	LOA (version not reported)
4. Alternate statistical methods adjusted using the mean of repeated measurements versus standard LOA					
Ambrosini et al.			Standard LOA, Pearson correlation coefficient (PCC); mean agreement		
Anvari et al.	Standard LOA		Standard LOA, Intraclass correlation coefficient (ICC)		
Batterham et al.			Standard LOA, Two one-sided t-test (TOST); paired t-test		
Euser et al.			Standard LOA, Coefficient of variation (CV)		
5. Alternate statistical methods unadjusted for repeated measurements versus standard LOA					
Armstrong et al.	Standard LOA, Pearson correlation coefficient (PCC); paired t-test	Standard LOA, Pearson correlation coefficient (PCC); paired t-test			
Bland & Altman, 1986 [3]	Standard LOA, Pearson correlation coefficient (PCC)				
Kim and Lee	Standard LOA; concordance correlation coefficient (CCC); Pearson correlation coefficient (PCC); bias correction factor				
Lorne et al.		Standard LOA, Interchangeability rate			
Rankin and Stokes	Standard LOA; ICC				
Saugel et al.	Standard LOA; error grid analysis (EGA)				
6. Adjustments for repeated measurements not reported for LOA estimates and alternate statistical methods					
Lombard et al.					LOA (version not reported), Spearman correlation; Wilcoxon signed-rank test; cross classification; weighted kappa

*Abbreviations:* *CCC* Concordance correlation coefficient, *CE* Coefficient of error, *CEIV* Correlated-errors-in-variables model, *CV* Coefficient of variation, *EGA* Error grid analysis, *GCI* Generalized confidence intervals, *HB1* Exchangeable multivariate hierarchical Bayesian model, *HB2* Non-exchangeable multivariate hierarchical Bayesian model, *ICC* Intraclass correlation coefficient, *LME* Linear mixed-effects model, *LOA* Limits of agreement, *PCC* Pearson correlation coefficient, *PE* Percentage error, *SEM* Structural equations model, *TDI* Total deviation index, *TOST* Two one-sided t-test

*Both unadjusted LOA and adjusted LOA for repeated measurements are presented in these papers for comparison purposes

**LOA data extracted from Bland & Altman articles

The first group (*n* = 4) presented their findings based on different methods to compute LOAs [1, 14–16]. Three of the four publications included results from a standard LOA analysis that considered all data as independent measures, and all three reported wider limits when using the adjusted LOA methods [1, 15, 16]. The authors of three of the four publications proposed novel or modified approaches to the LOA method, using a random effects model, reporting narrower limits of agreement with those, when compared with “fixed effect” adjusted LOAs [14–16].

The second group (*n* = 7) adjusted for the correlated nature of repeated measurements for all statistical methods presented, but did not compare different versions of the LOA methods [19, 21, 28–31, 35].

The third group (*n* = 7) presented an adjusted statistical approach for the analysis of method comparison studies with repeated measurements, but presented results from the standard LOA method [17, 18, 20, 23, 25, 26, 32]. Three did not directly report LOA findings and were extracted elsewhere [17, 25, 26], while two did not report whether LOA findings were adjusted and this could not be deduced from the publication [20, 32].

The fourth group (*n* = 4) generated the mean of repeated measurements, providing only one average measurement per participant, per method being compared [33, 34, 37, 39].

The fifth group (*n* = 6) used a dataset containing repeated measurements, but did not adjust the statistical analyses for repeated measurements [3, 22, 24, 27, 38, 40]. In three of those, repeated measurements were used to examine intra-rater reliability and the repeated measurements were excluded from the inter-rater reliability analysis, using only one of the measurements per individual [3, 38, 40]. Others did not intend to propose statistical methods to adjust for repeated measurements, but nevertheless used a dataset with repeated measurements. In those cases, only one measurement was chosen and the repeated measures were excluded from the analysis [22, 27], or the repeated measurements were all included in the analyses independently without being adjusted for their correlated nature [24, 38].

#### Reported strengths and limitations of the LOA method

Reported qualitative findings pertaining to the strengths and limitations of the LOA method are presented in Table 4. The most commonly reported strength (*n* = 9) was its ease of use and interpretation [1, 3, 16, 17, 26, 30, 32, 34, 39].

**Table 4:** Summary of strengths and limitations of the LOA methods, as reported by the authors of the included publications

Strengths	Limitations
Simple / straightforward / easy to do and interpret [1, 3, 16, 17, 26, 30, 32, 34, 39]	Assumptions about the normality and the homoscedasticity of the distribution of the data need to be met [1, 14, 16, 23, 24, 32, 35]
Provides information about the bias and magnitude of differences between methods [33, 36, 40]	Agreement limits are subjective and based on clinical judgement, not based on statistical factors [17, 33, 36, 37]
Provides a visual representation of the comparison between methods [23, 40]	Does not provide information about which method is superior [16, 17, 30]
Results are based on the original unit of measurement, allowing for direct comparison with a clinically-acceptable difference between methods [16]	There is uncertainty in point estimates that can lead to biased conclusions, 95% CI should be presented [16, 34]

The most commonly reported limitation (*n* = 7) was the parametric nature of this statistical method [1, 14, 16, 23, 24, 32, 35]. Others have indicated that LOA results do not provide information about which method is most accurate [16, 17, 30], which can be a limitation specifically in cases where the gold standard provides worse estimates than the newer method [17].

## Discussion

Our review identified multiple approaches to inform the analysis of method comparison studies with repeated measurements of continuous clinical data. Among 29 methodological publications included in this work, different approaches to handling repeated data were identified. Further, a variety of statistical methods were directly compared and reviewed for their strengths and limitations.

A critical distinction in method comparison studies is that between agreement and reliability (repeatability). Reliability measures, such as intraclass correlation coefficients or correlation-based metrics, quantify the consistency of measurements within a method (or between raters), but do not directly assess agreement between methods. High reliability can therefore coexist with poor agreement. In contrast, agreement-based approaches, such as limits of agreement, explicitly characterize the magnitude and clinical relevance of differences between methods. This distinction is particularly important in studies with repeated measurements, where within-subject variability constrains the maximum achievable agreement between methods. This conceptual distinction has been emphasized previously, including by Olofsen, who highlighted how reliance on reliability metrics in repeated-measurement method comparison studies can lead to misleading inferences about agreement [41].

Three main variants of the LOA method were identified, including the standard LOA method [1, 3] the LOA method adjusted for repeated measurements proposed by B&A^1,2^, and novel approaches to the LOA method, proposed by other authors, using random effects to allow for adjustment of other covariates and better generalizability [14–16]. Several authors emphasized the importance of adjusting for repeated measurements, considering the within-subject variability and thus yielding wider LOAs [1, 14–16]. B&A have recommended data visualization techniques to verify that data distributions respect the assumptions of normality and homoscedasticity [1, 2]. Other authors have proposed alternative statistical methods when those conditions are not met, such as Bayesian statistics [28], bias and precision plots [31, 32], probability of agreement [29], log-transformation of data [39], and regression-based extensions of the Bland–Altman approach to address non-uniform differences [1].

Thirty-two statistical methods were identified from the included publications, of which six generate agreement intervals that use the same unit of measurement as the outcome being measured, facilitating interpretation and comparison to the clinically acceptable difference [16]. A priori specification of clinically acceptable agreement thresholds is an essential prerequisite for meaningful agreement assessment. However, subjectivity arises primarily when such benchmarks are absent, poorly justified, or applied post hoc. Moreover, because limits of agreement are estimates, their associated uncertainty should be considered when evaluating whether agreement meets predefined clinical criteria. [16, 24]. Consistent with this, our review found that most studies compared estimated limits of agreement directly with predefined benchmarks, whereas only one study explicitly incorporated uncertainty around the limits of agreement—for example, through assessment of confidence bands, as proposed by Taffé [32].

The other statistical methods generate findings that do not have a unit of measurement, such as coefficients, which may lead to greater difficulty in interpretation [16]. While B&A have consistently recommended against the use of correlation coefficients and comparison of means [1, 3], this review found that these methods are often proposed for the analysis of method comparison studies. Some have promoted the use of correlation coefficients as an appropriate method to examine reliability [37] or to detect bias [38]. Others do not recommend the use of this approach given the fact that it measures association rather than agreement [3, 33, 36], and does not provide sufficient information to examine the level of agreement between methods [33, 36, 40]. Consistent with frameworks distinguishing absolute agreement from relative reliability, limits-of-agreement–based approaches quantify absolute differences between methods on the measurement scale of interest, whereas reliability metrics such as correlation coefficients and intraclass correlation coefficients assess relative consistency rather than interchangeability. In repeated-measurement designs, adjusted limits of agreement preserve this absolute interpretation while appropriately accounting for within- and between-subject variability.

Sample size also has an important influence on agreement assessment, irrespective of the statistical method used. Smaller studies yield less precise estimates of agreement, resulting in wider confidence intervals around limits of agreement and greater uncertainty when comparing results to predefined clinical benchmarks. Conversely, larger sample sizes improve precision but do not mitigate issues related to inappropriate choice of agreement or reliability metrics.

When planning the statistical analysis of a method comparison study with repeated measurements, it is critical to examine the type of repeated measurements to choose appropriate statistical approaches. This review has grouped included publications into five different categories to help readers identify sources of evidence that may be of value based on the planned approach to handling repeated measurements (Table 3). The publications included in the first three groups may assist in choosing alternate statistical methods that adjust for the correlation between repeated measurements and the data included in Table 2 provides information on the performance of these methods when compared with the LOA approach. However, prudence is recommended when comparing results obtained from each statistical method in the third group, as the LOA findings were not adjusted for repeated measurements.

The fourth and fifth groups of publications presented in Table 3 are alternative approaches that have been suggested for the analysis of method comparison studies. In the fourth group of publications, the means of repeated measurements were used. These cannot be recommended for general use, except perhaps in cases where this approach represents standard clinical practice [20, 42]. However, this approach is at risk of omitting important information about the distribution of the differences between methods [30]. Finally, the fifth group did not adjust for repeated measurements. Given the wide range of more robust statistical methods available to adjust for repeated measurements in method comparison studies, the exclusion of repeated measurements from analysis is not recommended.

Taken together, the agreement methods identified in this review can be broadly categorized into those that directly assess agreement and those that do not. Limits-of-agreement–based approaches, including standard and adjusted Bland–Altman methods and related interval-based extensions, quantify absolute differences between methods on the measurement scale of interest and are appropriate for assessing interchangeability. In contrast, statistical approaches such as correlation coefficients, paired t-tests, and Wilcoxon signed-rank tests assess association or mean differences rather than agreement and therefore do not provide sufficient information to evaluate agreement between methods. Although frequently used in practice, these latter approaches may lead to misleading conclusions when applied to method comparison studies.

To our knowledge, there are no other systematic reviews of methodological publications aiming at identifying and comparing statistical methods to inform the analysis of method comparison studies with repeated measurements. A systematic review examined 210 method comparison studies and reported that the LOA method is the statistical method that is most often used (85%), with the inappropriate use of correlation coefficients remaining in use by many authors (27%)^5^. That review did not focus on repeated measurements, as opposed to our review.

There are limitations to the current scoping review. The literature search was conducted up to December 2022, and it is therefore possible that more recent methodological publications were not captured. As statistical methodology continues to evolve, future updates may be required to incorporate newer approaches to agreement assessment in studies with repeated measurements. The LOA approach was used as an anchor for search, whereby only publications referring to “limits of agreement” or “Bland-Altman” within their abstracts were considered for inclusion, to allow for the comparison of findings between the LOA method and other statistical methods. It is possible this approach may have prevented the identification of other statistical methods that would have been appropriate for the analysis of method comparison studies with repeated measurements. Multiple versions of the LOA method exist in the literature, and the method adjusted for repeated measurements was not always used in included publications, limiting the quantitative comparisons of findings between different statistical methods. Finally, since methodological publications were specifically sought for, the aim of those papers was usually not to comment on the agreement between the methods in the context of the dataset used to examine the statistical methods, yielding limitations pertaining to the conclusions about agreement between methods.

## Conclusion

This scoping review synthesizes existing statistical approaches for the analysis of method comparison studies with repeated measurements of clinical data and summarizes how these methods perform relative to different versions of the limits-of-agreement approach. Overall, the findings suggest that adjusted limits of agreement are often more appropriate for repeated-measurement designs, as they account for within-subject variability while preserving an absolute interpretation of agreement.

## Supplementary Information

Supplementary Material 1.

## Data Availability

All data are presented in the main manuscript or additional supporting files.

## Abbreviations

B&A: Bland & Altman
LOA: Limits of agreement
